# Prevalence of Type-D Personality and Its Association with Pain, Disability, and Psychological Distress in a University Spine Outpatient Clinic: A Cross-Sectional Study

**DOI:** 10.3390/jcm15051753

**Published:** 2026-02-25

**Authors:** Christian Riediger, Mark Ferl, Christoph H. Lohmann, Maria Schönrogge, Agnieszka Halm-Pozniak

**Affiliations:** Orthopaedic University Clinic Magdeburg, Otto-von-Guericke-University Magdeburg, 39106 Magdeburg, Germany

**Keywords:** Type-D personality, chronic pain, spine disorders, psychological distress, outpatient care

## Abstract

**Objectives**: Type-D personality, characterized by negative affectivity (NA) and social inhibition (SI), has been associated with adverse outcomes in chronic pain and cardiovascular populations. Evidence in spine outpatient settings remains limited. We aimed to assess the prevalence of Type-D personality and its association with pain, disability, and psychological distress in patients presenting to a university spine outpatient clinic. **Methods**: This exploratory cross-sectional study included 300 consecutive patients (18–85 years) presenting to a university spine outpatient clinic between 2023 and 2025. Patients completed the Type-D Scale-14 (DS14; Type-D defined as NA ≥10 and SI ≥10), the Hospital Anxiety and Depression Scale (HADS), the Visual Analog Scale for pain (VAS, 0–10), and the Oswestry Disability Index (ODI, 0–100). Demographic and clinical characteristics were recorded. Comparisons between Type-D and non-Type-D patients were performed. **Results**: The prevalence of Type-D personality was 32.3% (95% CI: 27.0–37.6%). Compared with non-Type-D patients, Type-D patients reported higher pain intensity (VAS: 5.23 vs. 3.88), disability (ODI: 38.6 vs. 31.3), anxiety (HADS-A: 10.0 vs. 6.5), and depression (HADS-D: 8.4 vs. 6.4); all *p* < 0.01. Between-group differences were clinically relevant, with large effect sizes for pain intensity (VAS; Cohen’s d ≈ 1.10) and moderate-to-large effect sizes for functional disability (ODI; Cohen’s d ≈ 0.75). Correlation analyses showed moderate to strong associations between Type-D personality traits (negative affectivity and social inhibition) and psychological distress. In stratified analyses, longer pain duration was descriptively associated with greater disability, particularly among patients with Type-D personality. **Conclusions**: Type-D personality is common in spine outpatient populations and is associated with greater pain, disability, and psychological distress. These findings underscore the relevance of psychosocial factors in spine outpatient care and highlight the need for further longitudinal research to clarify prognostic implications and potential targets for intervention.

## 1. Introduction

Low back pain (LBP) is one of the most common musculoskeletal conditions worldwide and represents a leading cause of disability, with a lifetime prevalence approaching 70–80% in Western societies [1]. Global Burden of Disease analyses consistently rank LBP among the top causes of years lived with disability across all age groups [1,2]. Many patients presenting to spine outpatient clinics suffer from chronic or recurrent symptoms, while structural abnormalities on imaging often show weak correlations with reported pain intensity or functional impairment [2]. This discrepancy highlights the importance of psychosocial factors in shaping pain perception, illness behavior, and treatment response.

Among established psychosocial risk factors, maladaptive cognitions such as catastrophizing, fear-avoidance beliefs, reduced self-efficacy, and social stressors have been shown to contribute to pain chronification and poor functional recovery [3,4]. In accordance with the biopsychosocial model, pain is increasingly understood as a multidimensional experience influenced not only by nociceptive input but also by affective, cognitive, and behavioral processes [5]. Within this framework, stable personality traits have gained attention as predisposing factors that may influence coping strategies, symptom perception, and healthcare utilization [6].

Type-D personality, also referred to as the “distressed” personality type, represents a well-defined psychosocial construct characterized by high negative affectivity (NA) and high social inhibition (SI) [7]. Individuals with Type-D personality are prone to persistent emotional distress, social withdrawal, and impaired stress coping. Initially described in cardiovascular populations [7,8], Type-D personality has subsequently been associated with adverse outcomes across a broad range of medical conditions, including oncology, orthopedics, and pain medicine [3,4,5,6,8,9,10,11,12,13]. In musculoskeletal disorders, Type-D personality has been linked to higher pain intensity, increased disability, reduced quality of life, and an elevated risk of pain chronification [3,10,11,12,13].

Beyond behavioral and psychological mechanisms, emerging evidence suggests that Type-D personality may be associated with biological processes relevant to chronic pain, including autonomic dysregulation, altered hypothalamic–pituitary–adrenal axis activity (HPA), and inflammatory signaling [14,15,16,17,18]. These pathways overlap with mechanisms implicated in persistent musculoskeletal pain. Such biological mechanisms have not been directly assessed in the present study and should therefore be considered hypothesis-generating.

However, despite increasing interest in Type-D personality as a transdiagnostic psychosocial risk factor, its prevalence and clinical relevance in ambulatory spine outpatient populations remain insufficiently investigated. Most existing studies have focused on cardiovascular cohorts or postoperative spine surgery patients, in whom Type-D personality has been associated with delayed recovery and lower patient satisfaction [11,13,19,20].

Previous studies have examined Type-D personality in selected chronic pain populations, including patients with fibromyalgia and other rheumatologic conditions, demonstrating associations with psychological distress, reduced self-esteem, and impaired quality of life [11,13,20]. However, these studies were largely conducted in disease-specific cohorts with established chronic pain syndromes and often focused on psychological constructs rather than clinical pain- and disability-related outcomes in routine spine care.

In contrast, data on Type-D personality in unselected spine outpatient populations at the time of initial presentation prior to treatment stratification or surgical decision-making remain scarce. Spine outpatient clinics represent a heterogeneous patient population with varying pain durations, diagnoses, and treatment histories, in which psychosocial risk factors may play a critical yet underrecognized role in symptom burden and functional impairment.

Therefore, the present study aimed (i) to assess the prevalence of Type-D personality among patients attending a university spine outpatient clinic and (ii) to examine its association with pain intensity, functional disability, and psychological distress. By focusing on a real-world spine outpatient setting, the present study extends existing literature on Type-D personality beyond disease-specific chronic pain cohorts.

## 2. Methods

### 2.1. Study Design and Setting

This study was conducted as an exploratory cross-sectional observational study at the Orthopaedic University Spine Outpatient Clinic of Otto-von-Guericke University Magdeburg, Germany. Consecutive adult patients presenting for specialist evaluation of spine-related pain between September 2023 and September 2025 were considered for inclusion. The study was conducted in accordance with the Declaration of Helsinki and is reported in line with the Strengthening the Reporting of Observational Studies in Epidemiology (STROBE) guidelines for cross-sectional studies.

### 2.2. Participants

Adult patients aged 18 to 85 years presenting with cervical, thoracic, or lumbar spine-related pain were eligible for inclusion. Patients were included irrespective of pain duration or prior conservative treatment in order to reflect a real-world outpatient spine population. Pain duration was systematically assessed at baseline using a standardized patient intake questionnaire, in which patients reported the duration of their current spine-related pain symptoms in months. Pain duration was pre-specified as a clinically relevant variable prior to analysis and was included both as a covariate in regression models and as a stratification variable for exploratory subgroup analyses.

Exclusion criteria included insufficient German language proficiency to complete self-administered questionnaires, severe cognitive impairment precluding reliable questionnaire completion, and major psychiatric disorders. Severe cognitive impairment was assessed clinically by the treating physician based on medical history and current presentation. Major psychiatric disorders were defined as previously diagnosed psychotic disorders, bipolar disorder, or severe major depressive episodes requiring acute psychiatric treatment, as documented in the medical record or reported by the patient.

All eligible patients received written and oral information about the study and provided written informed consent prior to enrollment.

### 2.3. Assessment of Type-D Personality

Type-D personality was assessed using the validated 14-item Type-D Scale (DS14), which measures the two core dimensions negative affectivity (NA) and social inhibition (SI) [7]. Each subscale consists of seven items rated on a 5-point Likert scale, yielding subscale scores ranging from 0 to 28. In accordance with established criteria, patients scoring ≥10 on both NA and SI were classified as having a Type-D personality [7].

The DS14 has demonstrated good internal consistency, construct validity, and clinical applicability in cardiovascular, musculoskeletal, and chronic pain populations [5,7,10,11,12,13].

### 2.4. Clinical and Psychometric Outcomes

Pain intensity, functional disability, and psychological distress were assessed using standardized and validated instruments commonly applied in spine and pain research.

Pain intensity was measured using a Visual Analog Scale (VAS, 0–10), where 0 represents “no pain” and 10 represents “worst imaginable pain” [21].

Functional disability was assessed using the Oswestry Disability Index (ODI, 0–100), which quantifies spine-related limitations in activities of daily living, with higher scores indicating greater disability [22].

Psychological distress was evaluated using the Hospital Anxiety and Depression Scale (HADS), including the anxiety (HADS-A) and depression (HADS-D) subscales, each ranging from 0 to 21. The HADS has demonstrated good reliability and validity across clinical and musculoskeletal populations [23].

All questionnaires were completed by patients prior to physician consultation to minimize potential influence of clinical interaction on self-reported outcomes.

### 2.5. Additional Clinical Variables

Demographic and clinical variables were obtained from standardized intake forms and medical records. Collected variables included age, sex, body mass index (BMI), smoking status, relevant comorbidities (hypertension, diabetes mellitus, and previously diagnosed depression), primary spine diagnosis, pain duration, history of spine surgery, prior or ongoing physiotherapy, and analgesic medication use.

Primary spine diagnoses were assigned by the treating physician based on clinical examination and imaging findings and grouped into major diagnostic categories for descriptive analyses.

### 2.6. Statistical Analysis

Statistical analyses were performed using SPSS version 25.0 (IBM Corp. IBM SPSS Statistics for Windows, Version 25.0; IBM Corp.: Armonk, NY, USA, 2017).

Continuous variables are presented as means ± standard deviations, and categorical variables as frequencies and percentages. Normality of continuous variables was assessed by visual inspection of histograms and Q–Q plots and analytically using the Shapiro–Wilk test. Group comparisons between patients with and without Type-D personality were conducted using independent-samples *t*-tests or non-parametric equivalents, as appropriate. Categorical variables were compared using chi-square or Fisher’s exact tests.

Pearson correlation coefficients were calculated to explore associations between psychometric measures, including DS14 subscales, pain intensity (VAS), functional disability (ODI), and psychological distress (HADS-A and HADS-D) [5,23].

Multivariable linear regression analyses were performed to explore associations between Type-D personality and clinical outcomes (pain intensity and functional disability). Regression models were adjusted for age, sex, body mass index, pain duration, and relevant comorbidities. Results are reported as regression coefficients with 95% confidence intervals and corresponding *p*-values.

Given the exploratory nature of the study, no formal correction for multiple comparisons was applied. Results should therefore be interpreted with caution, particularly for secondary outcomes. Missing data were minimal (<5% for all variables) and were handled using complete-case analysis. A two-sided *p*-value <0.05 was considered statistically significant.

### 2.7. Sample Size Considerations

Given the observational and exploratory nature of the present cross-sectional study, no formal a priori sample size calculation was performed. However, a post hoc sensitivity analysis was conducted to evaluate the adequacy of the sample size to detect clinically meaningful between-group differences.

Assuming a two-sided alpha level of 0.05 and a statistical power of 80%, the available sample size of 300 patients (approximately 1:2 distribution of Type-D and non-Type-D personality) provided sufficient power to detect small-to-moderate effect sizes (Cohen’s d ≥ 0.35) in continuous outcome measures such as pain intensity and functional disability. Larger effect sizes, as observed in the present analyses, could be detected with substantially higher statistical power

### 2.8. Ethical Considerations

The study was conducted in accordance with the Declaration of Helsinki and received approval from the Ethics Committee of the Otto-von-Guericke University Magdeburg, Germany (approval numbers 30/22 and 172/15). Written informed consent was obtained from all participants prior to inclusion.

## 3. Results

### 3.1. Patient Characteristics

A total of 300 patients attending the university spine outpatient clinic were included in the analysis. The mean age of the study population was 55 ± 12 years, and 55% of patients were female. Based on DS14 criteria, 97 patients (32.3%; 95% CI: 27.0–37.6%) were classified as having a Type-D personality, while 203 patients (67.7%) were classified as non-Type-D. Although the predefined inclusion criteria allowed enrollment of adult patients aged 18 to 85 years, the observed age range differed between groups. Patients classified as having a Type-D personality included younger adults, whereas the non-Type-D group did not comprise patients below the age of 31 years. This difference reflects the consecutive real-world recruitment process of the outpatient clinic and the distribution of Type-D personality traits across age groups, rather than the application of any age-related exclusion criteria. Importantly, mean age did not differ significantly between groups.

Baseline demographic and clinical characteristics stratified by Type-D personality status are summarized in Table 1. There were no significant differences between groups with regard to age, sex distribution, or body mass index. The most frequent primary diagnoses in the overall cohort were lumbar spinal stenosis, lumbar disc herniation, and non-specific low back pain. The distribution of primary spine diagnoses did not differ significantly between patients with and without Type-D personality (χ^2^ test, *p* = 0.41; Table 2). A graphical overview of diagnostic distributions is provided in Supplementary Figure S1.

### 3.2. Prevalence of Type-D

**Figure 1:**
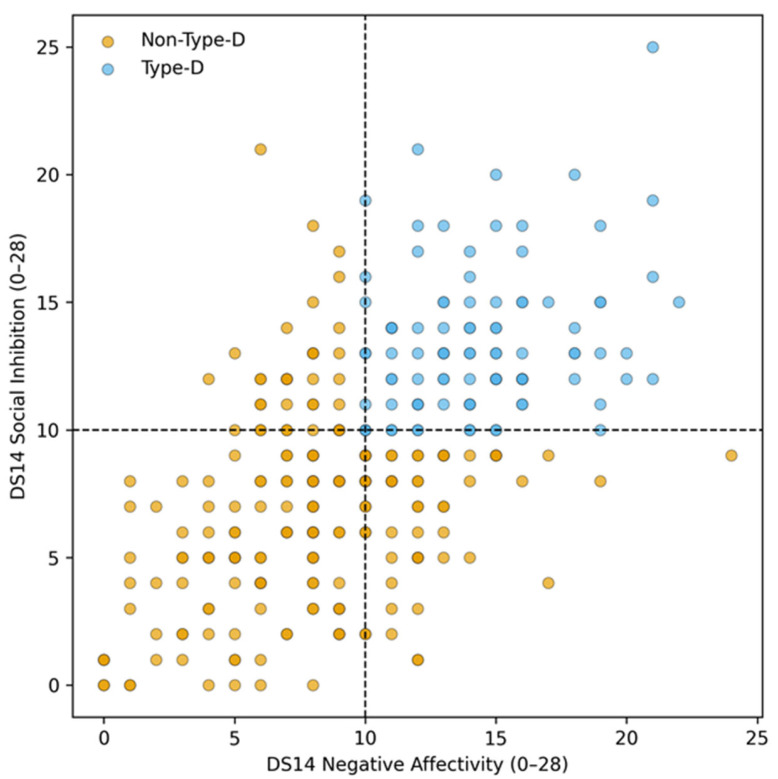
Distribution of DS14 negative affectivity and social inhibition scores. Scatter plot illustrating the distribution of DS14 negative affectivity (NA) and social inhibition (SI) scores assessed by the Type-D Scale-14 (DS14). Dashed lines indicate the established cut-off values (NA ≥10 and SI ≥10) used to classify Type-D personality. Patients meeting both criteria were classified as having a Type-D personality.

Type-D personality was identified in 32.3% (95% CI: 27.0–37.6%) of patients attending the spine outpatient clinic. The distribution of negative affectivity (NA) and social inhibition (SI) subscale scores and the applied classification thresholds are shown in Figure 1. NA and SI scores demonstrated a moderate-to-strong intercorrelation, consistent with the conceptual definition of the Type-D construct.

### 3.3. Pain Intensity, Functional Disability, and Psychological Distress

Group comparisons of pain intensity, functional disability, and psychological distress are summarized in Table 1. Patients with a Type-D personality reported significantly higher pain intensity as measured by the Visual Analog Scale (VAS) compared with non-Type-D patients (5.23 ± 1.3 vs. 3.88 ± 1.2, *p* < 0.001), corresponding to a large effect size (Cohen’s d ≈ 1.10). Functional disability assessed by the Oswestry Disability Index (ODI) was also significantly higher in the Type-D group (38.6 ± 10.2 vs. 31.3 ± 9.5, *p* = 0.002), with a moderate-to-large effect size (Cohen’s d ≈ 0.75).

**Figure 2:**
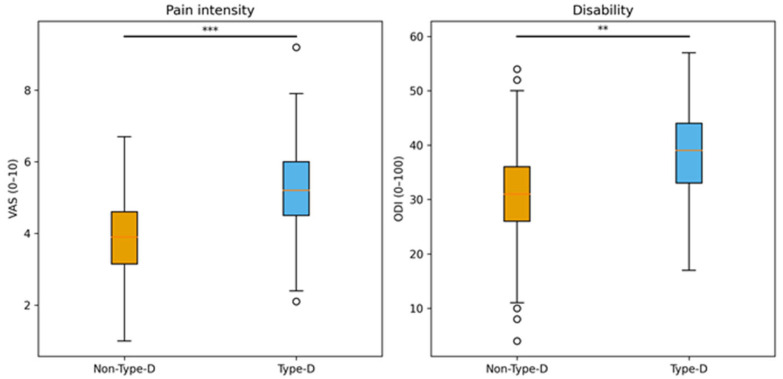
Pain intensity and functional disability by Type-D personality. Boxplots showing the distribution of pain intensity measured by the Visual Analog Scale (VAS) and functional disability assessed by the Oswestry Disability Index (ODI) in patients with and without Type-D personality. Boxes represent interquartile ranges (IQR), horizontal lines indicate medians, and whiskers extend to 1.5 times the interquartile range (IQR). Statistical significance between groups is indicated above each panel (** *p* < 0.01, *** *p* < 0.001).

Measures of psychological distress differed markedly between groups. Patients with Type-D personality showed higher anxiety scores on the HADS-A (10.0 ± 4.1 vs. 6.5 ± 3.9, *p* < 0.001) and higher depression scores on the HADS-D (8.4 ± 4.2 vs. 6.4 ± 3.8, *p* < 0.001). Group differences in pain intensity and functional disability are visualized in Figure 2.

### 3.4. Multivariable Regression Analyses

In multivariable linear regression analyses adjusted for age, sex, body mass index, pain duration, and documented depression diagnosis, Type-D personality remained significantly associated with higher pain intensity and greater functional disability. Specifically, Type-D personality was associated with an increase of 1.18 points in pain intensity on the Visual Analog Scale (VAS) (β = 1.18, 95% CI 0.92–1.44, *p* < 0.001). The overall model explained 29% of the variance in pain intensity (adjusted R^2^ = 0.27, *p* < 0.001). Similarly, Type-D personality was independently associated with an increase of 6.42 points in the Oswestry Disability Index (ODI) (β = 6.42, 95% CI 4.21–8.63, *p* < 0.001). The model accounted for 34% of the variance in functional disability (adjusted R^2^ = 0.32, *p* < 0.001). Complete regression results, including all covariates, are presented in Table 3.

### 3.5. Correlation Analyses

Pearson correlation analyses between psychometric measures are summarized in Table 4. A visual representation of correlations is shown in Supplementary Figure S2. Both DS14 subscales (negative affectivity and social inhibition) showed weak-to-moderate positive correlations with pain intensity (VAS), functional disability (ODI), and psychological distress (HADS-A and HADS-D). The strongest association was observed between negative affectivity and social inhibition (r = 0.58, *p* < 0.001), indicating substantial overlap between the two components of the Type-D construct. Given the approximately continuous nature of the psychometric scale scores, Pearson correlation coefficients were considered appropriate.

### 3.6. Pain and Disability Stratified by Pain Duration

**Figure 3:**
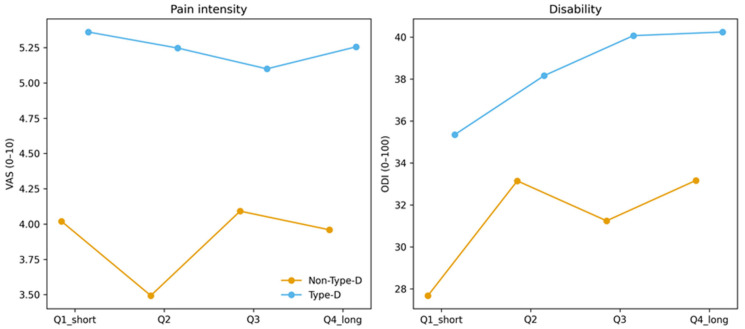
Pain intensity and functional disability stratified by pain duration and Type-D personality. Line plots illustrating mean pain intensity (VAS) and functional disability (ODI) across quartiles of pain duration in patients with and without Type-D personality. Values are presented descriptively to visualize trends across pain duration categories; no formal interaction testing was performed.

Mean pain intensity and functional disability across pain duration quartiles are presented descriptively in Table 5. Figure 3 illustrates descriptive trends of pain intensity and disability across pain duration quartiles. Across increasing pain duration quartiles, patients with Type-D personality consistently reported higher pain intensity (mean VAS) and greater disability (mean ODI) compared with non-Type-D patients. The absolute difference appeared most pronounced in the longest pain duration quartile; however, these analyses were descriptive and no formal interaction testing was performed.

## 4. Discussion

In this cross-sectional study, approximately one-third of patients presenting to a university spine outpatient clinic fulfilled criteria for Type-D personality. This prevalence aligns closely with previous studies which have reported comparable prevalence rates of Type-D personality in chronic pain and musculoskeletal populations [5,11,13]. Our findings indicate that Type-D personality is common in spine outpatient care and represents a clinically relevant psychosocial phenotype rather than an incidental psychological trait.

Patients with Type-D personality reported significantly higher pain intensity and functional disability, accompanied by markedly elevated anxiety and depressive symptoms. This pattern is consistent with a substantial body of literature demonstrating associations between Type-D personality, increased symptom burden, impaired quality of life, and poorer clinical outcomes across various medical conditions [8,9,12,14]. In musculoskeletal disorders specifically, Type-D personality has been linked to greater pain severity, functional limitations, and psychological distress, supporting its relevance within the biopsychosocial model of pain [5,10,11,12,13,20].

### 4.1. Comparison with Previous Literature

Our findings are consistent with previous studies in orthopedic and spine-related populations showing that Type-D personality is associated with higher pain intensity and disability in conditions such as chronic low back pain, fibromyalgia, and upper extremity musculoskeletal disorders [5,10,11,12,13,20]. In surgical spine cohorts, Type-D personality has been linked to poorer postoperative recovery, increased complication rates, and lower patient satisfaction [24,25].

Importantly, most existing evidence originates from postoperative, disease-specific, or highly selected cohorts. In contrast, the present study extends these observations to an unselected, real-world spine outpatient population at the time of initial presentation. This highlights that the adverse associations of Type-D personality with pain, disability, and psychological distress are observed at the time of initial presentation in the clinical care pathway and are not restricted to advanced disease stages or surgical contexts.

### 4.2. Potential Mechanisms

Several psychological and biological mechanisms may contribute to the observed associations between Type-D personality and adverse pain-related outcomes. Psychologically, individuals with Type-D personality are characterized by high negative affectivity and social inhibition, traits that have been associated with maladaptive coping strategies such as catastrophizing, fear-avoidance behavior, and reduced help-seeking [3,4,26]. These factors are well-established contributors to pain chronification and functional disability in musculoskeletal disorders [5,6,10,18,27].

From a biological perspective, Type-D personality has been associated with autonomic nervous system dysregulation, including increased sympathetic activity, reduced parasympathetic (vagal) tone, and elevated inflammatory markers [8,14,28,29,30]. These alterations overlap with mechanisms implicated in central sensitization and persistent pain syndromes. While causal pathways cannot be inferred from the present data, the interaction between psychological vulnerability and biological stress responses represents a plausible explanatory framework.

These considerations should be regarded as hypothesis-generating, as no physiological or biological markers were assessed in the present study.

### 4.3. Clinical Implications

The present findings have relevant implications for clinical practice in spine outpatient care by highlighting the potential importance of psychosocial factors, including personality traits, in patients presenting with spine-related pain. Routine assessment of psychosocial risk factors may support a more comprehensive biopsychosocial characterization and early risk stratification. The DS14 questionnaire represents a brief, validated, and easily implementable screening tool that can be incorporated into clinical workflows without substantial burden [5,7,31]. However, it should be emphasized that the present cross-sectional study does not allow conclusions regarding the predictive validity of Type-D personality for long-term outcomes or treatment response.

Patients identified as having a Type-D personality may exhibit a higher symptom burden and psychological distress, which could justify consideration of multimodal management approaches integrating psychological interventions such as cognitive behavioral therapy, stress management, or mindfulness-based strategies alongside standard orthopedic care [32,33]. Importantly, the effectiveness of such approaches specifically in spine outpatient populations with Type-D personality has not yet been established and requires confirmation in prospective interventional studies.

In addition, emerging neuromodulatory approaches targeting autonomic regulation have gained increasing interest. Preliminary evidence suggests that modulation of vagal activity may influence pain perception and emotional regulation in chronic pain conditions [15,16,17,34]. Nevertheless, clinical data in spine outpatient populations remain limited, and such approaches should be regarded as investigational. These approaches should therefore be regarded as hypothesis-generating and warrant investigation in future longitudinal and interventional studies. Further longitudinal and interventional studies are necessary to determine whether identification of Type-D personality can inform targeted treatment strategies and improve clinical outcomes in this population.

### 4.4. Strengths and Limitations

Strengths of this study include the investigation of a consecutive, real-world spine outpatient cohort and the use of standardized, validated assessment instruments. Several limitations should be acknowledged.

First, the study was conducted at a single tertiary care university spine center, which may introduce selection bias. Patients referred to tertiary outpatient clinics may present with higher symptom burden, longer pain duration, or more complex clinical profiles than those managed in primary or secondary care settings, potentially limiting generalizability.

Second, the assessment relied predominantly on self-reported measures of pain, disability, and psychological distress. Objective clinical parameters, such as standardized physical examination findings or imaging characteristics, were not systematically incorporated into the analyses. Consequently, the relationship between Type-D personality and subjective symptom burden could not be contextualized against structural pathology or objective functional impairment.

Third, the absence of a comparison with general population norms limits the interpretation of the observed prevalence of Type-D personality and psychological distress. While prevalence estimates were compared with previously published clinical cohorts, direct comparisons with population-based reference samples were not feasible within the present study design.

Fourth, although no a priori sample size calculation was performed, post hoc sensitivity analyses indicated that the available sample size was sufficient to detect clinically relevant effect sizes in the primary outcomes. Nevertheless, the absence of an a priori power calculation should be considered when interpreting the results.

Fifth, given the number of statistical comparisons performed across multiple outcome measures, the risk of Type I error inflation cannot be excluded. As no formal correction for multiple comparisons was applied, findings particularly for secondary outcomes should be interpreted with appropriate caution.

Finally, conceptual overlap between the constructs assessed by the DS14 (negative affectivity) and the Hospital Anxiety and Depression Scale cannot be entirely excluded. Although these instruments capture related aspects of emotional distress, they represent distinct theoretical constructs: stable personality traits versus current symptom states. Some degree of shared variance may therefore have influenced the observed associations.

### 4.5. Future Directions

Future longitudinal studies are needed to determine whether Type-D personality predicts long-term pain trajectories, treatment response, and healthcare utilization in spine outpatient populations. The integration of objective physiological markers, such as heart rate variability or inflammatory biomarkers, may further elucidate underlying mechanisms [8,14]. Randomized controlled trials should evaluate whether targeted psychosocial or neuromodulatory interventions improve outcomes in patients with Type-D personality.

## 5. Conclusions

Type-D personality was present in approximately one-third of patients attending a university spine outpatient clinic and was associated with higher pain intensity, functional disability, and psychological distress. These findings underscore the clinical relevance of psychosocial factors in spine outpatient care and support the consideration of routine psychosocial screening to identify patients with an increased symptom burden at an early stage. While causal relationships cannot be inferred from this cross-sectional study, assessment of Type-D personality may contribute to a more comprehensive biopsychosocial characterization of patients in clinical practice. Future longitudinal studies are required to clarify the prognostic relevance of Type-D personality and to determine whether targeted psychosocial interventions can improve outcomes in this patient subgroup.

## Figures and Tables

**Table 1:** Baseline characteristics by Type-D personality status.

Variable	Non-Type-D (n = 203)	Type-D (n = 97)	*p*-Value
Age, years	55.4 ± 11.9	54.8 ± 12.2	0.68
Female sex, n (%)	114 (56.2)	51 (52.6)	0.56
Body mass index, kg/m^2^	27.4 ± 4.3	27.7 ± 4.9	0.61
Pain intensity (VAS)	3.88 ± 1.2	5.23 ± 1.3	<0.001
Disability (ODI)	31.3 ± 9.5	38.6 ± 10.2	0.002
Anxiety (HADS-A)	6.5 ± 3.9	10.0 ± 4.1	<0.001
Depression (HADS-D)	6.4 ± 3.8	8.4 ± 4.2	<0.001

Legend: Values are presented as mean ± standard deviation or number (%). Abbreviations: BMI, body mass index; VAS, Visual Analog Scale; ODI, Oswestry Disability Index; HADS-A/D, Hospital Anxiety and Depression Scale.

**Table 2:** Primary diagnoses by Type-D status.

Primary Diagnosis	Non-Type-D (n = 203)	Type-D (n = 97)
Cervical radiculopathy	18 (8.9%)	12 (12.4%)
Degenerative spondylolisthesis	28 (13.8%)	22 (22.7%)
Lumbar disc herniation	45 (22.2%)	21 (21.6%)
Lumbar spinal stenosis	75 (36.9%)	23 (23.7%)
Nonspecific low back pain	37 (18.2%)	19 (19.6%)
**Overall distribution (χ^2^ test)**		***p*** ** = 0.41**

Legend: Values are presented as number (%). Group differences were assessed using the chi-square test.

**Table 3:** Multivariable linear regression analyses examining the association between Type-D personality and (Model 1) pain intensity (VAS, 0–10) and (Model 2) functional disability (ODI, 0–100), adjusted for demographic and clinical covariates.

Model 1: Pain Intensity (VAS, 0–10)			
Model statistics: R^2^ = 0.29; Adjusted R^2^ = 0.27; F (6, 293) = 19.8; *p* < 0.001			
**Predictor**	**β (Unstandardized)**	**95% CI**	***p*** **-Value**
Type-D personality (yes vs. no)	1.18	0.92–1.44	<0.001
Age (years)	0.01	−0.01–0.02	0.31
Female sex (yes vs. no)	0.12	−0.15–0.39	0.38
Body mass index (kg/m^2^)	0.03	−0.01–0.07	0.14
Pain duration (months)	0.02	0.01–0.03	0.002
Depression diagnosis (yes vs. no)	0.46	0.12–0.80	0.008
**Model 2: Functional Disability (ODI, 0–100)**			
Model statistics: R^2^ = 0.34; Adjusted R^2^ = 0.32; F (6, 293) = 24.7; *p* < 0.001			
**Predictor**	**β (Unstandardized)**	**95% CI**	***p*** **-Value**
Type-D personality (yes vs. no)	6.42	4.21–8.63	<0.001
Age (years)	0.08	−0.05–0.21	0.22
Female sex (yes vs. no)	1.54	−1.22–4.31	0.27
Body mass index (kg/m^2^)	0.41	0.08–0.74	0.015
Pain duration (months)	0.18	0.11–0.25	<0.001
Depression diagnosis (yes vs. no)	3.26	1.14–5.39	0.003

Legend: Unstandardized regression coefficients (β) with 95% confidence intervals (CI) are presented. Type-D personality (yes vs. no) was entered as the primary predictor. All models were adjusted for age, sex, body mass index (BMI), pain duration (months), and documented depression diagnosis. R^2^ and adjusted R^2^ indicate explained variance of the full model. VAS, Visual Analog Scale; ODI, Oswestry Disability Index; CI, confidence interval.

**Table 4:** Correlation matrix of psychometric scales.

Variable	VAS	ODI	HADS-A	HADS-D	DS14-NA	DS14-SI
VAS	1.00	0.27 (<0.001)	0.16 (0.006)	0.08 (0.17)	0.27 (<0.001)	0.34 (<0.001)
ODI	0.27 (<0.001)	1.00	0.11 (0.06)	0.10 (0.09)	0.21 (<0.001)	0.16 (0.005)
HADS-A	0.16 (0.006)	0.11 (0.06)	1.00	0.15 (0.01)	0.21 (<0.001)	0.27 (<0.001)
HADS-D	0.08 (0.17)	0.10 (0.09)	0.15 (0.01)	1.00	0.09 (0.12)	0.10 (0.08)
DS14-NA	0.27 (<0.001)	0.21 (<0.001)	0.21 (<0.001)	0.09 (0.12)	1.00	0.58 (<0.001)
DS14-SI	0.34 (<0.001)	0.16 (0.005)	0.27 (<0.001)	0.10 (0.08)	0.58 (<0.001)	1.00

Legend: Pearson correlation coefficients (r) with corresponding two-sided *p*-values are shown. Abbreviations: VAS, Visual Analog Scale; ODI, Oswestry Disability Index; HADS-A/D, Hospital Anxiety and Depression Scale (Anxiety/Depression); DS14-NA, negative affectivity; DS14-SI, social inhibition.

**Table 5:** Pain intensity and disability stratified by pain duration quartiles and Type-D status.

Pain Duration Quartile	Mean VAS—Non-Type-D	Mean VAS—Type-D	Mean ODI—Non-Type-D	Mean ODI—Type-D
Q1 (shortest duration)	3.78	4.96	31.98	33.85
Q2	3.80	5.21	33.76	35.10
Q3	3.83	4.84	31.74	36.06
Q4 (longest duration)	4.22	5.54	32.94	41.46

Legend: Mean pain intensity (VAS) and functional disability (ODI) are shown across quartiles of pain duration, stratified by Type-D personality status. Values are presented as mean values. Analyses are descriptive; no measures of variability or formal statistical testing were applied for pain duration strata.

## Data Availability

The data supporting the findings of this study are available from the corresponding author upon reasonable request. Due to privacy and ethical restrictions, data are not publicly available.

## Supplementary Materials

The following supporting information can be downloaded at: https://www.mdpi.com/article/10.3390/jcm15051753/s1, Figure S1. Distribution of primary spine diagnoses by Type-D personality. Bar Bar chart showing the number of patients with and without Type-D personality across major spine diagnostic categories. The figure provides a graphical overview of diagnostic distributions summarized numerically in Table 2; Figure S2. Correlation heatmap of psychometric measures. Heatmap illustrating Pearson correlation coefficients between pain intensity (VAS), functional disability (ODI), anxiety (HADS-A), depression (HADS-D), negative affectivity (DS14-NA), and social inhibition (DS14-SI). Exact correlation coefficients and corresponding p-values are provided in Table 3.

## Abbreviations

BMI	Body Mass Index
DS14	Denollet Scale, 14-item questionnaire for Type-D personality
HADS	Hospital Anxiety and Depression Scale
HADS-A	Hospital Anxiety and Depression Scale, Anxiety subscale
HADS-D	Hospital Anxiety and Depression Scale, Depression subscale
HPA	Hypothalamic–Pituitary–Adrenal (axis)
IQR	Interquartile Range
LBP	Low Back Pain
NA	Negative Affectivity
ODI	Oswestry Disability Index
SI	Social Inhibition
VAS	Visual Analog Scale
