# Supplementary material for: Prevalence of Type-D Personality and Its Association with Pain, Disability, and Psychological Distress in a University Spine Outpatient Clinic: A Cross-Sectional Study
Source: J Clin Med. 2026 Feb 25;15(5):1753. doi: 10.3390/jcm15051753 (PMC12985532; doi:10.3390/jcm15051753)
Supplement: Supplementary file 1 [file jcm-15-01753-s001.zip › jcm-4127154-supplementary.pdf]

Supplementary Materials

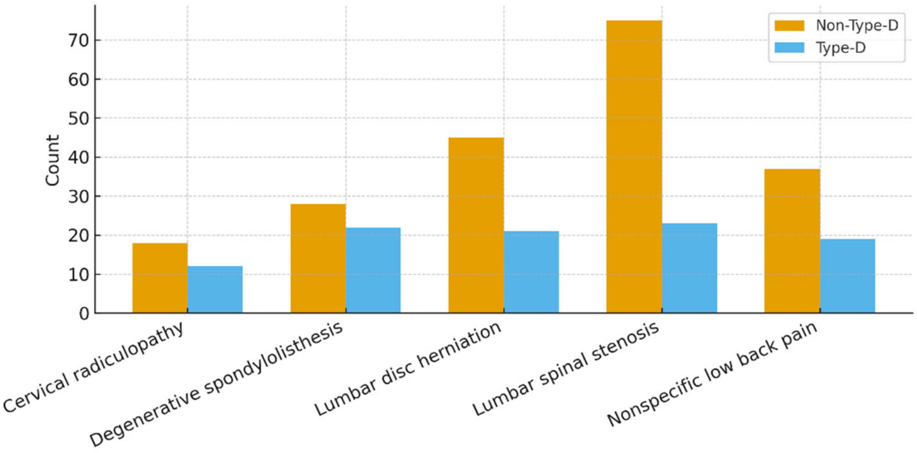

Figure S1. Distribution of primary spine diagnoses by Type-D personality. Bar Bar chart showing the number of patients with and without Type-D personality across major spine diagnostic categories. The figure provides a graphical overview of diagnostic distributions summarized numerically in Table 2.

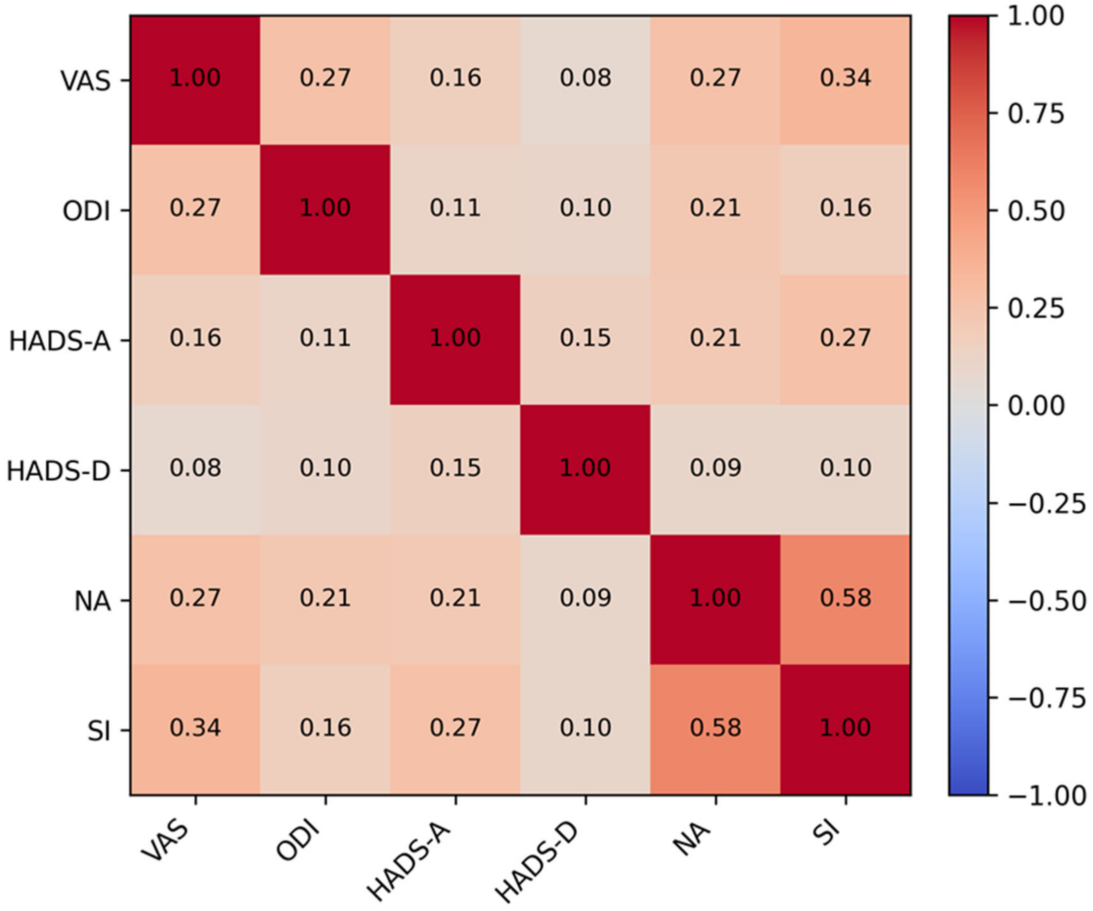

Figure S2. Correlation heatmap of psychometric measures. Heatmap illustrating Pearson correlation coefficients between pain intensity (VAS), functional disability (ODI), anxiety (HADS-A), depression (HADS-D), negative affectivity (DS14-NA), and social inhibition (DS14-SI). Exact correlation coefficients and corresponding p-values are provided in Table 3.
